# Connecticut Valley Dental Association

**Published:** 1872-12

**Authors:** L. C. Taylor


### Connecticut Valley Dental Association.

At the tenth annual meeting of this Society the following were were elected officers for the ensuing year: President—Dr. J. Searle Hurlburt; First Vice President—Dr. E. S. Davenport; Second Vice President—Dr. H. M. Miller; Secretary—L. C. Taylor; Treasurer—E. M. Goodrich; Executive Committee—Dr. F. Searle, Prof. L. D. Shepard, Dr. J. F. Adams.

President Riggs' retiring address was upon the subject of "General Dentistry."

He urged upon the profession the importance of being thorough in all examinations and operations; also the special importance of keeping the mouth clean, which can with difficulty be done, unless the teeth are in a regular position.

Article 2d—"The Use and Abuse of the Mallet," was taken up. By request of Prof. Shepard, that a general expression might be had, it was discused by each member in his turn, with the following results.

Dr. Searle opened the discussion, strongly advocating conservative practice—believing patients prefer hand pressure. Followed by Drs. Harwood, Bishop, and Goodrich, who think the abuse of the mallet has done away with its use. Drs. Clapp, Noble, Shepard, Morgan, Howland, Cowan, Jones, Riggs, Miller, Murilless, and many others, use the mallet, with hand pressure, believing the judicious use of the two forces, will produce better results than can be obtained by the radical use of either Dr. E. S. Davenport claims to have replaced many fillings, securing good results with the mallet where he had failed with hand pressure.

### EVENING SESSION.

The presiding officer being absent, Dr. Anderson was called to the chair.

Article 3d. "The use of the wedge, the file, and the chisel" was discused, each having advocates, whilst the latter was claimed to possess many advantages when used according to Dr. Arthur's theory.

Article 4th. "Mack's Retaining Screws" were strongly urged upon the profession by Drs. Mills, and Shepard, who believe them to be the only source of success in many difficult cases.

Article 5th. "Comparative success with other materials than vulcanite as a base for artificial teeth." Several specimens of celluloid and coralline base were presented; and the opinion was advanced that the further perfection of one or both materials would produce a base with qualities superior to rubber.

SECOND DAY.

President Hurlbut in the chair.

Subject 1—"Regulating Teeth," was taken from the table and introduced with an essay, by L. C. Taylor, on "Special Orthodontia," followed by Dr. Riggs, who presented casts, representing a case of a patient sixteen years of age, recently treated by him, where the six year molars were extracted to gain room to carry all the anterior teeth back, at the same time spreading them sufficiently to admit the two centrals to drop into the regular position, thereby depressing them more than one-half their diameters. Prof. Shepard also presented casts, with very interesting remarks; claiming that the law of forces must be thoroughly understood before difficult cases can be treated successfully. At the same time condemning the practice of extracting the cuspids, especially where the six year molars have been previously removed, as was shown by casts presented, which leave the patient with a deformed expression for life.

After three hours of very interesting discussion, adjourned to meet at Worcester, June, 1873.

L. C. Taylor, Sec.

